# Association of body mass index and physical activity with fatigue, depression, and anxiety among Iranian patients with multiple sclerosis

**DOI:** 10.3389/fneur.2023.1126215

**Published:** 2023-04-13

**Authors:** Nasim Rezaeimanesh, Pegah Rafiee, Roghayyeh Saeedi, Sharareh Eskandarieh, Mohammad Ali Sahraian, Pegah Khosravian, Maryam Abolhasani, Soodeh Razeghi Jahromi, Abdorreza Naser Moghadasi

**Affiliations:** ^1^Department of Clinical Nutrition and Dietetics, Faculty of Nutrition Sciences and Food Technology, National Nutrition and Food Technology Research Institute, Shahid Beheshti University of Medical Sciences, Tehran, Iran; ^2^Multiple Sclerosis Research Center, Neuroscience Institute, Tehran University of Medical Sciences, Tehran, Iran; ^3^Medical Plants Research Center, Basic Health Sciences Institute, Shahrekord University of Medical Sciences, Shahrekord, Iran; ^4^Sports Medicine Research Center, Neuroscience Institute, Tehran University of Medical Sciences, Tehran, Iran

**Keywords:** multiple sclerosis, fatigue, depression, anxiety, physical activity, body mass index

## Abstract

**Introduction:**

Depression, fatigue, and anxiety are three common clinical comorbidities of multiple sclerosis (MS). We investigated the role of physical activity (PA) level and body mass index (BMI) as modifiable lifestyle factors in these three comorbidities.

**Methods:**

A cross-sectional study was conducted in the MS specialist clinic of Sina Hospital, Tehran, Iran. Demographic and clinical data were collected. BMI was categorized in accordance with the WHO’s standard classification. Physical activity (PA) level and sitting time per day were obtained using the short form of the International Physical Activity Questionnaire (IPAQ-SF). Fatigue, anxiety, and depression scores were measured using the Persian version of the Fatigue Severity Scale (FSS), Beck Anxiety Inventory (BAI), and Beck’s Depression Inventory II (BDI-II) questionnaires, respectively. The correlation between the metabolic equivalent of tasks (MET), BMI, and daily sitting hours with depression, anxiety, and fatigue were checked using the linear regression test. The normal BMI group was considered a reference, and the difference in quantitative variables between the reference and the other groups was assessed using an independent sample t-test. Physical activity was classified with tertiles, and the difference in depression, anxiety, and fatigue between the PA groups was evaluated by a one-way ANOVA test.

**Results:**

In total, 85 MS patients were recruited for the study. The mean ± SD age of the participants was 39.07 ± 8.84 years, and 72.9% (*n*: 62) of them were female. The fatigue score was directly correlated with BMI (*P*: 0.03; *r*: 0.23) and sitting hours per day (*P*: 0.01; *r*: 0.26) and indirectly correlated with PA level (*P* < 0.01; *r*: −0.33). Higher depression scores were significantly correlated with elevated daily sitting hours (*P*: 0.01; *r*: 0.27). However, the correlation between depression with PA and BMI was not meaningful (*p* > 0.05). Higher anxiety scores were correlated with BMI (*P*: 0.01; *r*: 0.27) and lower PA (*P*: 0.01; *r*: −0.26). The correlation between anxiety and sitting hours per day was not significant (*p* > 0.05). Patients in the type I obesity group had significantly higher depression scores than the normal weight group (23.67 ± 2.30 vs. 14.05 ± 9.12; *P*: 0.001). Fatigue (32.61 ± 14.18 vs. 52.40 ± 12.42; *P*: <0.01) and anxiety (14.66 ± 9.68 vs. 27.80 ± 15.48; *P*: 0.01) scores were significantly greater among participants in the type II obesity group in comparison with the normal weight group. Fatigue (*P*: 0.01) and anxiety (*P*: 0.03) scores were significantly different in the three levels of PA, but no significant difference was found in the depression score (*P*: 0.17).

**Conclusion:**

Our data suggest that a physically active lifestyle and being in the normal weight category are possible factors that lead to lower depression, fatigue, and anxiety in patients with MS.

## Introduction

There is a reported prevalence of 2.5 million cases of multiple sclerosis (MS) worldwide (1). The estimated point prevalence of MS in Tehran, the capital city of Iran, was 167.54 individuals per 100.000 in 2020. Moreover, according to the increasing trend of MS prevalence, it is predicted that it will reach 220.84 per 100.000 individuals in 2029 (2). While the disease can begin at any age, the most common age of onset is between 20 and 40 years old (1).

MS patients may present various symptoms, including cognitive, psychiatric, sensory, motor, urinary, and vision, which may affect their quality of life. Depression, fatigue, and anxiety are three typical clinical manifestations of motor and psychiatric comorbidities, and their treatment has not been well established until now (1, 3). The prevalence of depression and anxiety in MS patients is reported to be 27.01 and 35.19%, respectively, and for fatigue, the prevalence ranges from 36.5 to 78.0% in different studies (3, 4). Anxiety and depression affect the occupational and social aspects of an MS patient’s life (3). On the other hand, fatigue may impact the patient’s capacity to work, employment status, and sick leave (4). MS mainly occurs among the young working-age population and could affect different aspects of their lives through its symptoms and comorbidities. Thus, many physicians use symptom therapies to improve MS patients’ quality of life.

To date, MS has no definite treatment. Hence, studies focusing on the modifiable environmental or lifestyle risk factors of MS or its comorbidities are highly regarded. Weight and physical activity are two samples of lifestyle factors. Obesity and low physical activity are linked with the risk of very chronic disorders through the inflammatory pathways and multiple sclerosis (5–8).

The available evidence has mentioned the association between obesity and higher clinical disability in MS patients (9). Still, data on the association between obesity and depression, fatigue, and anxiety scores among MS patients are controversial (10, 11).

Unfortunately, disability in movement, fear regarding safety, reluctance to participate in activities, and doubt in the ability to engage in physical activities, etc., cause significantly lower activity in MS patients compared with the healthy population (12). However, it seems that PA could have beneficial effects on physical symptoms and cognitive ability and improve the quality of life of MS patients (13). On the other hand, until now, only a few studies have investigated the correlation between the physical activity level of patients with MS and their scores of depression, fatigue, and anxiety, which are contentious too (11, 14–16).

Due to the impacts of MS comorbidities on the various aspects of patients’ lives that result in a decreasing quality of life (4), the increasing prevalence of MS worldwide (17), and the lack of definite treatment for these comorbidities (17), it is necessary to investigate the factors affecting these common comorbidities to improve patients’ quality of life. PA and BMI are modifiable lifestyle factors, and data on the association between them and depression, fatigue, and anxiety among MS patients are modest and inconsistent (10, 11, 14–16). To our knowledge, no study has mentioned the correlation between daily sitting hours and these comorbidities. Therefore, in the present study, we aimed to investigate the association of BMI, PA, and sitting time per day with depression, fatigue, and anxiety among Iranian patients with MS.

## Materials and methods

### Study population

A cross-sectional study was performed in the MS specialist clinic of Sina Hospital, Tehran, Iran, from December 2021 to September 2022. In total, 85 patients with MS among the patients referred to this clinic during the study period were interested in participating in the study, met the inclusion criteria, and were enrolled.

The definite diagnosis of MS was performed according to the revised McDonald criteria 2017 (18) and neurologist confirmation. The inclusion criteria were as follows: age between 18 and 55 years old; Expanded Disability Status Scale (EDSS) score at less than 5.5; educational status of diploma or higher; not having any other neurological disorder apart from MS; not having other chronic diseases such as chronic gastrointestinal, liver, kidney, heart, respiratory, and cancer diseases; not receiving treatment for depression; not being on a special diet; more than 1 year since diagnosis; more than 1 month since the last corticosteroid pulse therapy and the last relapse; and not being pregnant or lactating.

### Patients’ consent and protocol approval

The study method and aims were described for the participants, and they themselves completed the written consent. The study protocol was reviewed and approved by the ethics committee of the Shahid Beheshti University of Medical Science under the ethics number: IR.SBMU.nnftri.Rec.1400.096.

### Data collection

Demographic and clinical data were collected by a personal information form during in-person interviews. The Expanded Disability Status Scale (EDSS) score was calculated by an expert neurologist during the patients’ examination. Weight and height were obtained using a Seka scale with minimal clothing and an accuracy of 100 grams and a tape measure with an accuracy of 0.5 cm, respectively. BMI was calculated by the weight (kg)/[height (m)]^2^ formula. BMI was categorized considering the World Health Organization’s (WHO) standard classification of underweight (BMI < 18.5), normal weight (BMI ≥ 18.5 and < 25), overweight (BMI ≥ 25 and < 30), obesity type I (BMI ≥ 30 and < 35), and obesity type II (BMI ≥ 35) (19).

The International Physical Activity Questionnaire–Short Form (IPAQ-SF) was used to assess physical activity levels and sitting hours per day (20). The metabolic equivalent of tasks (MET) was calculated using the frequency and duration of physical activity and was reported as MET-min/wk. (21).

Fatigue, anxiety, and depression scores were measured using the Persian version of the Fatigue Severity Scale (FSS) (22), Beck Anxiety Inventory (BAI) (23), and Beck’s Depression Inventory II (BDI-II) (24) questionnaires, respectively.

BDI-II is a 21-item self-administered multiple-choice questionnaire that measures depression severity over the past 2 weeks. Each of the 21 symptoms is rated from 0 to 1, and a total score is obtained from the summation of all items (25). The scoring cut points used according to the manual for the BDI–II are as follows: 0–13 minimal range, 14–19 mild, 20–28 moderate, and 29–63 severe.

FSS is a nine-item scale in which each item rates between 1 = strongly disagree and 7 = strongly agree and a total score of 9 to 63, and it assesses fatigue intensity over the past 2 weeks. The higher the FSS score, the more severe the fatigue. The considered cut-off values were as follows: <36 = no-to-mild fatigue, 36–52 = moderate fatigue, and >52 = severe fatigue (26).

BAI refers to the 21 clinical symptoms of cognitive and physical anxiety experienced by the participants over the last week. The respondent can choose one of the four options of “not at all: 0,” “mild: 1,” “moderate: 2,” and “severe: 3” for each item. The total score may range from 0 to 63: 0–7 (minimal anxiety level), 8–15 (mild anxiety), 16–25 (moderate anxiety), and 26–63 (severe anxiety) (27, 28).

### Statistical analysis

The data were analyzed using SPSS 26 software. The normal distribution of variables was examined by the Kolmogorov–Smirnov test. The correlation between MET, BMI, and sitting hours per day with depression, anxiety, and fatigue were checked using the linear regression test. Further analysis was conducted by adjusting the linear regression model for age, gender, marital and employment status, educational level, cigarette smoking and alcohol consumption status, MS type, MS drug, disease duration, EDSS, and relapse rate. The normal BMI group was considered a reference, and the difference in quantitative variables between the reference and each of the other groups was assessed using the independent sample *t*-test. Physical activity was classified with tertiles and labeled low, medium, and high physical activity. The difference in depression, anxiety, and fatigue between PA groups was evaluated with a one-way ANOVA test. A value of *p* of less than 0.05 was considered to be statistically significant. For the ANOVA *post hoc*, *P* < 0.016 was considered statistically significant considering the Bonferroni correction.

## Results

In total, 85 MS patients were recruited for the present study. The mean ± SD age of participants was 39.07 ± 8.84 years old, and 72.9% (*n*: 62) of them were women. A total of 62.4% (*n*: 53) of the patients suffered from relapsing–remitting MS (RRMS), 32.9% (*n*: 28) had secondary progressive MS (SPMS), and the others had the primary progressive type of MS. The mean ± SD of EDSS, annual attack rate, and disease duration of the patients were 3.1 ± 1.72, 0.7 ± 1.4, and 11.8 ± 6.3 years, respectively. The delay between the first onset of MS and the definite diagnosis was 0.92 ± 2.14 years. A total of 57 (67.1%) participants were married, and 22 (25.9%) were single; 38.8% (*n*: 33) had bachelor’s degrees, and 34.1% (*n*: 29) had diploma degrees; 42 (49.4%) were unemployed, and 78.5% (*n*: 33) were housekeepers; 16 (18.8%) were cigarette smokers, the same number as alcohol consumers. Most of the participants were receiving ocrelizumab (*n*: 55; 64.7%) as MS treatment, followed by natalizumab (*n*: 25; 29.4%), fingolimod (*n*: 3; 3.5%), rituximab (*n*: 1; 1.2%), and dimethyl fumarate (*n*: 1; 1.2%) (Table 1).

**Table 1:** Baseline characteristics of participants.

Variables		
Female sex^&^		62 (72.9%)
Age (years)^$^		39.07 (8.84)
EDSS^$^		3.01 (1.72)
Annual attack rate (number)^$^		0.7 (1.4)
Time passed from onset (years)^$^		11.8 (6.3)
Time passed from diagnosis (years)^$^		10.8 (5.8)
MS type^&^	RRMS	53 (62.4%)
	SPMS	28 (32.9%)
	PPMS	4 (4.7%)
Treatment ^&^	Ocrelizumab	55 (64.7%)
	Natalizumab	25 (29.4%)
	Rituximab	1 (1.2%)
	Fingolimod	3 (3.5%)
	Dimethyl fumarate	1 (1.2%)
Marital status^&^	Single	22 (25.9%)
	Married	57 (67.1%)
	Divorced	3 (3.5%)
	Death of wife/husband	3 (3.5%)
Education^&^	Diploma	29 (34.1%)
	Associate degree	10 (11.8%)
	Bachelor	33 (38.8%)
	Master of sciences	10 (11.8%)
	PhD or higher	3 (3.5%)
Employment status^&^	Employment	43 (50.6%)
	Unemployment	42 (49.4%)
Cigarette smoker^&^	Yes	16 (18.8%)
	No	69 (81.2%)
Alcohol consumer^&^	Yes	16 (18.8%)
	No	69 (81.2%)

& These data are presented as number (percent).

$ These data are presented as mean (standard deviation).

EDSS, Expanded Disability Status Scale. RRMS, Relapsing remitting MS. SPMS, Secondary progressive MS. PPMS, Primary progressive MS. Annual attack rate for RRMS patients.

As demonstrated in Table 2, the study participants had a mean ± SD BMI, MET, and sitting hours per day of 24.9 ± 4.8 kg/m^2^, 918.9 ± 1359.4 MET-min/wk., and 5.6 ± 3.2 h, respectively. The largest number of patients was in the mild fatigue (*n*: 37; 43.5%), minimal depression (*n*: 35; 41.2%), and mild anxiety (*n*: 26; 30.6%) categories. More details on depression, fatigue, and anxiety categorization and the mean ± SD are also reported in this table.

**Table 2:** Descriptive statistics of participants.

Variables		Mean (SD)/*N* (%)
BMI (kg/m^2^)		24.9 (4.8)
MET (MET-min/wk)		918.9 (1359.4)
Sitting hours per day		5.6 (3.2)
Fatigue score		36.9 (15.2)
Fatigue	Mild	37 (43.5%)	Moderate	34 (40.0%)
Severe	14 (16.5%)
Depression score		16.3 (10.0)
Depression	Minimal	35 (41.2%)	Mild	20 (23.5%)
Moderate	17 (20.0%)
Severe	13 (15.3%)
Anxiety score		16.5 (10.6)
Anxiety	Minimal	19 (22.4%)	Mild	26 (30.6%)
Moderate	21 (24.7%)
Severe	19 (22.4%)

MET: Metabolic equivalent of tasks. Fatigue, anxiety, and depression scores were assessed using Fatigue Severity Scale (FSS), Beck Anxiety Inventory (BAI), and Beck’s Depression Inventory II (BDI-II) questionnaires, respectively. Data are presented as mean (standard deviation).

Table 3 presents the correlation of modifiable lifestyle factors, including BMI, PA level, and sitting time per day, with depression, anxiety, and fatigue in MS patients. Higher BMI (*P* unadjusted: 0.03, *r*: 0.23; *P* adjusted: 0.03, *r*: 0.26) and sitting time (*P* unadjusted: 0.01, *r*: 0.26; *P* adjusted: 0.01, *r*: 0.31) showed a significant direct correlation with fatigue. Meanwhile, this significant correlation was indirect in the case of the PA level and fatigue (*P* unadjusted <0.01, *r*: −0.33; *P* adjusted: 0.03, *r*: −0.25). Even though the depression score directly correlated with BMI and demonstrated an adverse correlation with the PA level, these correlations were not significant (*p* > 0.05). Higher sitting time per day was correlated with a higher depression score (*P* unadjusted: 0.01, *r*: 0.27; *P* adjusted<0.01, *r*: 0.31). The analysis regarding anxiety indicated that higher BMI (*P* unadjusted: 0.01, *r*: 0.27; *P* adjusted<0.01, *r*: 0.37) and sitting time per day (*P* adjusted: 0.03, *r*: 0.26) as well as a lower PA level (*P* unadjusted: 0.01, *r*: −0.26) are correlated with higher anxiety among MS patients.

**Table 3:** The correlation of body mass index, physical activity, and sitting hours per day with fatigue, depression, and anxiety.

		BMI	MET	Sitting hours/day
Fatigue score	Unadjusted model^&^	0.03 (0.23)	**<0.01 (−0.33)**	**0.01 (0.26)**
	Adjusted model*	**0.03 (0.26)**	**0.03 (−0.25)**	**0.01 (0.31)**
Depression score	Unadjusted model^&^	0.45 (0.08)	0.09 (−0.18)	**0.01 (0.27)**
	Adjusted model*	0.39 (0.10)	0.12 (−0.17)	**<0.01 (0.31)**
Anxiety score	Unadjusted model^&^	**0.01 (0.27)**	**0.01 (−0.26)**	0.14 (0.16)
	Adjusted model*	**<0.01 (0.37)**	0.24 (−0.14)	**0.03 (0.26)**

Data are presented as value of *p* (beta), calculated by linear regression analysis.

& *p*-value < 0.016 considered significant by applying Bonferroni correction for unadjusted model (0.05/3 = 0.016).

* Linear regression model adjusted for age, gender, marital and employment status, educational level, cigarette smoking and alcohol consumption status, MS type, MS drug, disease duration, EDSS, and relapse rate.

Fatigue, anxiety, and depression scores were assessed using Fatigue Severity Scale (FSS), Beck Anxiety Inventory (BAI), and Beck’s Depression Inventory II (BDI-II) questionnaires, respectively. MET, Metabolic equivalent of tasks. The bold values are statistically significant.

The differences in depression, fatigue, and anxiety scores between the normal weight group of BMI and other categories are highlighted in Table 4. The patients in the type I obesity group had significantly higher depression scores than those in the normal weight group (23.67 ± 2.30 vs. 14.05 ± 9.12; *P*: 0.001). Fatigue (32.61 ± 14.18 vs. 52.40 ± 12.42; *P*: <0.01) and anxiety (14.66 ± 9.68 vs. 27.80 ± 15.48; *P*: 0.01) scores were significantly greater among participants in the type II obesity group in comparison with the normal weight group.

**Table 4:** The difference in fatigue, anxiety, and depression scores between BMI categories.

Variables	BMI						
	Normal weight BMI ≥ 18.5 and < 25 *n*: 41	Overweight BMI ≥ 25 and < 30 *n*: 32	Type I obesity BMI ≥ 30 and < 35 *n*: 3	Type II obesity BMI ≥ 35 *n*: 5	*P* ^1^	*P* ^2^	*P* ^3^
Depression	14.05 ± 9.12	17.32 ± 11.58	23.67 ± 2.30	19.00 ± 9.24	0.19	**0.001**	0.26
Fatigue	32.61 ± 14.18	39.19 ± 15.59	36.67 ± 19.29	52.40 ± 12.42	0.06	0.64	**<0.01**
Anxiety	14.66 ± 9.68	16.34 ± 10.35	25.33 ± 11.59	27.80 ± 15.48	0.48	0.07	**0.01**

Data are presented as mean ± standard deviation (SD). Fatigue, anxiety, and depression scores were assessed using Fatigue Severity Scale (FSS), Beck Anxiety Inventory (BAI), and Beck’s Depression Inventory II (BDI-II) questionnaires, respectively. *P* was calculated using two-sample *t*-test (normal weight is considered as reference group and the other groups are compared with this reference category). *P*^1^: Comparison of normal and overweight groups; *P*^2^: Comparison of normal weight and obesity type I groups; *P*^2^: Comparison of normal weight and obesity type II groups. The bold values are statistically significant.

Table 5 reports the differences in depression, fatigue, and anxiety scores between PA-level stratifications. The fatigue (*P*: 0.01) and anxiety (*P*: 0.03) scores were significantly different in the three levels of PA, but no significant difference was found in the depression score (*P*: 0.17). The mean ± SD of the fatigue score at the low PA level was 42.42 ± 16.15 vs. 30.71 ± 12.32 at the high PA level (*P*: 0.01).

**Table 5:** The difference in fatigue, anxiety, and depression scores between physical activity categories.

Variables	Physical activity (PA)						
	Low Range: 0.0–208.5 MET-min/wk *n*: 28	Moderate Range: 226.0–782.0 MET-min/wk *n*: 28	High Range: 815.0–8106.0 MET-min/wk *n*: 28	*P*	*P* ^1^	*P* ^2^	*P* ^3^
Depression	18.38 ± 10.47	17.18 ± 10.96	13.44 ± 8.36	0.17	0.89	0.17	0.35
Fatigue	42.42 ± 16.15	38.11 ± 15.31	30.71 ± 12.32	**0.01**	0.58	**0.01**	0.14
Anxiety	18.50 ± 10.94	18.86 ± 11.16	12.39 ± 8.88	**0.03**	0.99	0.08	0.05

Data are presented as mean ± standard deviation (SD). Fatigue, anxiety, and depression scores were assessed using Fatigue Severity Scale (FSS), Beck Anxiety Inventory (BAI), and Beck’s Depression Inventory II (BDI-II) questionnaires, respectively. *P* was calculated using one-way ANOVA test. *P*^1^: Comparison of low and moderate PA groups. *P*^2^: Comparison of low and high PA groups. *P*^2^: Comparison of moderate and high PA groups (*p* values are calculated using Tukey’s test, post hoc analysis). *P* < 0.016 is considered statistically significant considering Bonferroni correction. The bold values are statistically significant.

**GrapH 1:**
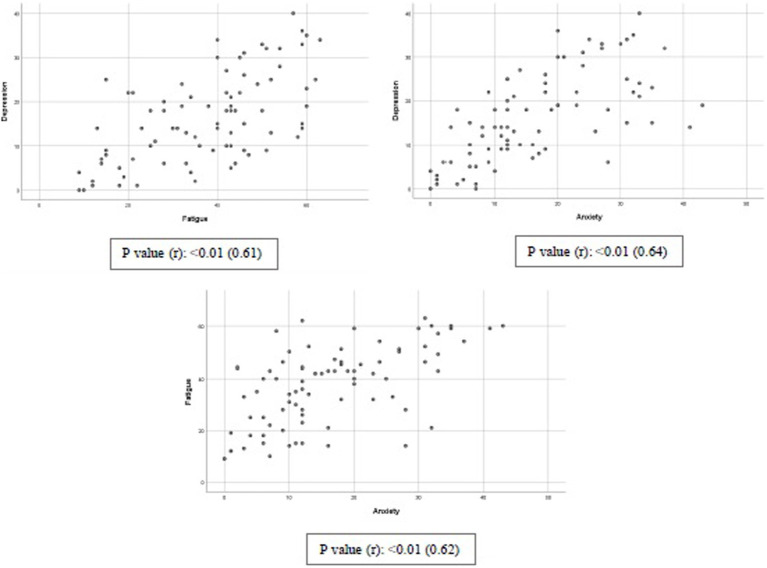


The correlation between depression, anxiety, and fatigue is reported in Graph 1. Significant direct correlations were found for depression and fatigue (*P*: <0.0; *r*: 0.61), depression and anxiety (*P*: <0.0; *r*: 0.64), and fatigue and anxiety (*P*: <0.0; *r*: 0.61).

## Discussion

In the present study, we investigated three modifiable lifestyle factors (BMI, PA, and sitting time per day) as influencing factors on three common comorbidities of MS, including depression, fatigue, and anxiety. Our data suggest that a lower BMI (i.e., having a normal BMI) may have protective effects on depression, fatigue, and anxiety. Being physically active is associated with lower fatigue and anxiety, and higher daily sitting hours are related to higher depression scores and more fatigue. These results support the importance of weight control in MS patients to improve their symptoms and quality of life.

We reported a correlation of lower physical activity and higher sitting time per day with some MS comorbidities, but the cause and effect are not clear in this correlation. Although depression, fatigue, and movement disability are among the barriers to physical activity in MS patients (12), investigations have highlighted the beneficial effects of physical activity and exercise on depression, fatigue, and anxiety improvement (29). In 2020, the US National MS Society mentioned the safety of exercise and lifestyle physical activity for MS patients, approved the beneficial effects of physical activity for disease, management of symptoms, overall health, and quality of life of MS patients, and provided physical activity and exercise recommendations for patients with different physical abilities from EDSS 0 to 9 (12). Neurologists and other healthcare providers could use this recommendation based on patients’ abilities, needs, and preferences.

Being overweight and obese are common comorbidities of MS that could be associated with low physical activity and a sedentary lifestyle (30). In other words, one of the effective strategies for weight loss and combating the secondary effects of obesity in MS patients is increasing the level of physical activity (30).

Both obesity and physical inactivity are reported as MS risk factors (8, 30). One proposed mechanism for preventing the effects of physical activity for MS risk is causing an increment in T regulatory cells, a decrease in the secretion of immunoglobulin, a shift of the Th1/Th2 balance, and a reduction in inflammation (8). In addition, exercise could protect MS by modulating immune factors and stress hormones (8). It seems the underlying mechanism in the association between obesity and elevated risk of MS refers to a significant increase in leptin level as an adipose tissue hormone that has a role in adaptive and innate immunity (31). Furthermore, a higher number of interleukin (IL)-6-, IL-17-, IL-2-, IL-15-, TNF-α-, and IFN-γ-producing cells is reported in overweight and obese MS patients compared to patients with normal weight. Increased production of these inflammatory cytokines is mentioned in the presence of leptin (31). In a recent study, the elevation of pro-inflammatory molecules such as IL-6 and leptins, as well as a reduction in anti-inflammatory cytokines such as IL-13, has been shown in the central nervous system (CNS) of obese MS patients (31).

The role of obesity and low physical activity in increasing inflammation has been proven in the general population and MS patients (6, 8, 32). On the other hand, it seems that one of the proposed mechanisms for depression, anxiety, and fatigue in MS patients is cytokine storm and the peripheral and central inflammatory and immunological pathways (33–35). Therefore, it could be concluded that elevated inflammation resulting from obesity and physical inactivity is the mediating mechanism of their correlation with depression, fatigue, and anxiety.

There is a hypothesis on the interrelationship between anxiety, fatigue, and depression among MS patients. Anxiety is a powerful factor in the development of depression among MS patients, and it is recommended for the initial assessment of anxiety to prevent and diagnose depression (36). In addition, it seems that depression is highly correlated with some other comorbidities of MS, including fatigue; thus, successful treatment of depression could result in a reduction in fatigue in patients with MS (35). Our results confirm these interactions, and we reported significant two-by-two correlations of depression, anxiety, and fatigue. It may be assumed that finding an approach to improve or prevent any of these may lead to the improvement of the others. Our study recommends having a healthy lifestyle, including increasing physical activity, lowering sitting time per day, and having a weight in the normal BMI range, to improve depression, anxiety, and fatigue in MS patients.

While the research regarding the effects of physical exercise on depression, fatigue, and anxiety is modest, most of the available studies report the beneficial role of higher physical activity in improving depression and fatigue in patients with MS, in line with our results (37, 38). Existing data on anxiety are controversial (16). In addition, increasing physical activity with an effect on weight loss may have additional beneficial effects on improving MS comorbidities. Therefore, recommending an active lifestyle may be beneficial to increase the quality of life of MS patients.

According to the American College of Sports Medicine Position Statement guideline, which seems to be useful for MS patients, individuals are recommended to engage in “150–250 min per week of moderate-intensity physical activity for preventing weight gain and reducing the risk of chronic diseases, and between 225 and 420 min per week of moderate-intensity physical activity for weight loss” (30).

## Conclusion

Considering the reduction in quality of life of MS patients and the impact of the disease on various dimensions of their personal, social, and occupational lives, studies on modifiable lifestyle factors in MS patients to increase their quality of life have been of great interest in recent years. The present study suggests a healthy lifestyle, which consists of having a normal weight and an active lifestyle (lower sitting hours per day and higher physical activity) as correlating factors to improve the motor and psychiatric comorbidities of MS and eventually improve the patients’ quality of life.

## Limitation

No blood samples were collected in this study, and the correlation of pro-inflammatory mediators was not investigated. It appears to be necessary to carry out future studies to find the mediating mechanisms of the observed relationships.

The current study is a cross-sectional study that only shows the relationship between the variables, and the exposure and outcome are unknown. Therefore, it is suggested that clinical studies should be designed and implemented to more closely examine the effectiveness of increasing physical activity and weight control on addressing the comorbidities of MS.

## Data availability statement

The raw data supporting the conclusions of this article will be made available by the authors, without undue reservation.

## Ethics statement

The studies involving human participants were reviewed and approved by Shahid Beheshti University of Medical Science by the ethic number: IR.SBMU.nnftri.Rec.1400.096. The patients/participants provided their written informed consent to participate in this study.

## Author contributions

NR: hypothesis, data collection, methodology, data entry and analysis, manuscript writing, and manuscript reviewing. PR: data collection, data entry, and manuscript reviewing. RS: methodology and data collection. SE: methodology, analysis, and manuscript reviewing. MS: supervision, data collection, and manuscript reviewing. PK: methodology and manuscript reviewing. MA: data collection and manuscript reviewing. SR: hypothesis, supervision, methodology, data collection, and manuscript writing and reviewing. AN: hypothesis, supervision, methodology, data collection, and manuscript writing and reviewing. All authors contributed to the article and approved the submitted version.

## Funding

This study was funded by the Shahid Beheshti University of Medical Sciences, grant number 29983.

## Conflict of interest

The authors declare that the research was conducted in the absence of any commercial or financial relationships that could be construed as a potential conflict of interest.

## Publisher’s note

All claims expressed in this article are solely those of the authors and do not necessarily represent those of their affiliated organizations, or those of the publisher, the editors and the reviewers. Any product that may be evaluated in this article, or claim that may be made by its manufacturer, is not guaranteed or endorsed by the publisher.
